# Orexin and MCH neurons: regulators of sleep and metabolism

**DOI:** 10.3389/fnins.2023.1230428

**Published:** 2023-08-22

**Authors:** Hanan Bouâouda, Pawan Kumar Jha

**Affiliations:** ^1^Pharmacology Institute, Medical Faculty Heidelberg, Heidelberg University, Heidelberg, Germany; ^2^Department of Systems Pharmacology and Translational Therapeutics, Perelman School of Medicine, University of Pennsylvania, Philadelphia, PA, United States

**Keywords:** sleep, metabolism, orexin, MCH, feeding, wake, lateral hypothalamus

## Abstract

Sleep-wake and fasting-feeding are tightly coupled behavioral states that require coordination between several brain regions. The mammalian lateral hypothalamus (LH) is a functionally and anatomically complex brain region harboring heterogeneous cell populations that regulate sleep, feeding, and energy metabolism. Significant attempts were made to understand the cellular and circuit bases of LH actions. Rapid advancements in genetic and electrophysiological manipulation help to understand the role of discrete LH cell populations. The opposing action of LH orexin/hypocretin and melanin-concentrating hormone (MCH) neurons on metabolic sensing and sleep-wake regulation make them the candidate to explore in detail. This review surveys the molecular, genetic, and neuronal components of orexin and MCH signaling in the regulation of sleep and metabolism.

## Introduction

Living beings on Earth maintain internal stability despite enormous environmental challenges. This process of maintenance of physiological stability is called homeostasis. In the mammalian body, homeostasis applies to the processes that regulate critical physiological parameters such as blood pressure, heart rate, plasma glucose, body temperature, feeding, and sleep. Feeding and sleep are mutually exclusive behaviors requiring distinct but interdependent homeostatic needs. To survive, organisms require strong coordination of these behaviors to achieve their respective homeostatic conditions. For example, wakefulness is required for foraging and food consumption. The mammalian hypothalamus crucially regulates these homeostatic functions.

The hypothalamus is one of the most complex and heterogeneous brain structures involved in the regulation of numerous homeostatic functions by integrating peripheral and central signals of circadian rhythms, sleep pressure, and energy metabolism. This diverse region of the brain is subdivided into 11 anatomically distinct nuclei having 34 neuronal and 11 non-neuronal cell types, cumulative actions of these cells regulate the sleep and metabolic processes (Chen et al., 2017; Rossi et al., 2019; Jha et al., 2022; Bear et al., 2023). The complex neuronal network made up of the projections from these nuclei to the entire brain, including intrahypothalamic connections regulates behavior and physiology. The metabolic aberrations in disturbed sleep conditions and the prevalence of sleep abnormalities in metabolic syndrome indicate the involvement of proximal hypothalamic neuronal circuitry regulating sleep and metabolism. The mechanistic understanding of these networks would be essential and bring wide-ranging clinical significance.

## Hypothalamic regulation of sleep and metabolism

The interactive action of arousal and sleep-promoting areas of the mammalian brain involves the regulation of the sleep-wake cycle (Saper and Fuller, 2017). Wake-sleep transition is the manifestation of inhibition of sleep or wake-promoting areas in the brainstem and hypothalamus (Saper, 2006). The pathway that stimulates and maintains wakefulness consists of glutamatergic inputs from parabrachial and pedunculopontine tegmental nuclei (PPT) to the basal forebrain (BF), and GABAergic and cholinergic neurons in the BF that innervates the cerebral cortex (Saper and Fuller, 2017). Further, GABAergic neurons in the lateral hypothalamus (LH) promote wakefulness by inhibiting sleep-promoting neurons in the thalamus and preoptic area (Herrera et al., 2016; Venner et al., 2016). Wake-promoting orexinergic neurons in the LH that mainly use glutamate to transmit their signals are spatially intermingled with sleep-promoting melanin-concentrating-hormone (MCH)-expressing cells (Rosin et al., 2003). The MCH-expressing cells are primarily GABAergic and found in LH and Zona Incerta (ZI) (Adamantidis and de Lecea, 2008b; Rolls et al., 2010). The projections of both orexin and MCH-expressing neurons to the cortex, hippocampus, amygdala, nucleus accumbens (NAc), hypothalamus, thalamus, ventral tegmental area (VTA), locus coeruleus (LC), and raphe nucleus indicating their intra- and extrahypothalamic functions (Concetti and Burdakov, 2021). Wake promotion inhibits sleep-promoting circuitries lie mainly in the hypothalamic ventrolateral preoptic (VLPO) and median preoptic (MnPO) areas. Sleep-active GABAergic neurons of preoptic areas and brainstem project to the wake-promoting area and inhibiting them in regulated manners (Saper and Fuller, 2017). This mutual inhibition regulates the sleep-wake transition.

Another critical role of the hypothalamus is to maintain energy homeostasis by regulating food intake. Like sleep, energy metabolism is also regulated by mutual inhibitory circuitries. The arcuate nucleus (ARC) harbors appetite-promoting Neuropeptide Y (NPY) and Agouti-related protein (AgRP) neurons that mutually inhibit the appetite-suppressing pro-opiomelanocortin (POMC) and amphetamine-related transcript (CART) neurons. These sets of neurons act as sensors of satiety-promoting leptin and appetite-stimulating ghrelin hormones. Leptins inhibit NPY/AgRP neurons and activate POMC/CART whereas ghrelin activates NPY/AgRP and inhibit POMC/CART neurons. The ARC integrates these peripheral signals and transmits them to other hypothalamic areas such as the dorsomedial nucleus (DMH), the paraventricular nucleus (PVH), and the LH (Milbank and Lopez, 2019). The orexin neurons in LH sense peripheral hormonal signals and levels of metabolites like glucose and amino acids. This is evident from the anatomical connection of orexin neurons to the other metabolic nuclei of the hypothalamus. Further, electrophysiological studies reveal that wake-promoting orexin neurons functionally regulate the NPY, POMC, and glucose-responsive neurons in the ARC and ventromedial nucleus of the hypothalamus (VMH) (Muroya et al., 2004). Interestingly, sleep-promoting neurons also regulate metabolism as fasting increases the expression of MCH levels, and activation of MCH neurons reduces energy expenditures (Pissios et al., 2006).

The LH is the heterogeneous structure located in the posterior hypothalamus and its diverse cell populations have been implicated in the regulation of an array of fundamental physiological processes that includes sleep, feeding, and energy metabolism (Stuber and Wise, 2016). The LH harbors heterogeneous neuronal subtypes including orexin, MCH, GABA, glutamatergic, galanin, neurotensin-releasing (Nts), leptin-receptor (LepRb) expressing neurons, and substance P-releasing neurons (Mickelsen et al., 2019; Rossi et al., 2019). In this review, we discuss the LH’s orexin and MCH neuronal circuitries that regulate energy metabolism and sleep.

## LH_MCH_ neurons

Melanin-concentrating hormone neurons are abundant in the LH, though few cells are located within the ZI. The LH_MCH_ neurons were reported to send extensive projections to different brain areas (Skofitsch et al., 1985; Bittencourt et al., 1992; Risold et al., 1997; Murray et al., 2000; Bittencourt, 2011), including structures involved in regulating sleep-wake cycle, feeding behavior, body weight and energy balance (Qu et al., 1996; Shimada et al., 1998; Verret et al., 2003; Jego et al., 2013; Konadhode et al., 2013; Yoon and Lee, 2013). Besides MCH peptide, LH_MCH_ neurons co-express many other neurotransmitters and neuropeptides including CART (Broberger, 1999; Elias et al., 2001), nesfatin-1 (Fort et al., 2008), neuropeptide-EI and neuropeptide-GE (Nahon et al., 1989; Bittencourt, 2011). To date, the molecular phenotypes of LH_MCH_ neurons is still a matter of debate to decipher whether these neurons co-release GABA, glutamate, or both. Indeed, previous studies have demonstrated that LH_MCH_ neurons express glutamic acid decarboxylase (GAD) 67 and GAD65 a key enzyme in GABA synthesis (Elias et al., 2001; Harthoorn et al., 2005; Shin et al., 2007). In addition, the results from the immunohistochemical study have reported that LH_MCH_ terminals express the vesicular GABA transporter (VGAT) that plays an essential role in carrying GABA from the neuronal cytoplasm into the synaptic cleft (Del Cid-Pellitero and Jones, 2012). In contrast, consecutive work showed that LH_MCH_ neurons do not overlap with VGAT-positive GABAergic neurons and were not able to express VGAT (Chee et al., 2015; Jennings et al., 2015; Mickelsen et al., 2017). Furthermore, Jego et al. (2013) confirmed that LH_MCH_ neurons release the inhibitory neurotransmitter GABA. Collectively, these findings hint that LH_MCH_ neurons may perhaps synthesize and release GABA. Paradoxically, other studies revealed that LH_MCH_ neurons are not exclusively GABAergic, but they also express the vesicular glutamate transporter 2 (VGLUT2) and presumably might produce glutamate (Abrahamson et al., 2001; Chee et al., 2015). More recently, by using molecular profiling including RNAscope combined with the immunohistochemical approach Schneeberger et al. (2018) highlighted that 97% of LH_MCH_ neurons express VGLUT2 but not VGAT suggesting that the vast majority of LH_MCH_ neurons are glutamatergic.

## LH_MCH_ neurons and energy metabolism

The role of LH_MCH_ neurons in the regulation of energy balance and metabolism has been studied vastly (Pissios et al., 2006; Al-Massadi et al., 2021; Lord et al., 2021). Earlier studies combing electrical stimulation and lesion approaches distinguished LH as a feeding center (Anand and Brobeck, 1951; Delgado and Anand, 1953; Teitelbaum and Stellar, 1954). More precisely, it was shown that LH_MCH_ neurons were directly involved in the modulation of energy balance and glucose homeostasis by controlling feeding behavior, adipose tissue thermogenesis, and locomotor activity. Under fasting conditions, MCH mRNA expression increased in lean mice as well as in leptin-deficient (*ob*/*ob*) obese mice (Qu et al., 1996). Acute intracerebroventricular (ICV) administration of MCH in rodents induced short-term but robust increase in food intake (Qu et al., 1996; Sahu, 1998; Della-Zuana et al., 2002) and chronic infusion enhances food consumption and body weight associated with the substantial increase in energy storage and reduction in energy expenditure (Della-Zuana et al., 2002; Gomori et al., 2003; Ito et al., 2003; Glick et al., 2009). Further insights supporting the critical role of LH_MCH_ neurons in regulation of energy homeostasis have emerged from genetic studies. Transgenic mice that overexpress MCH showed sustained hyperphagia, and mild weight gain associated with impaired glucose tolerance and insulin resistance (Ludwig et al., 2001). In contrast, targeted deletion of the *Mch* gene and MCH neurons-ablation exhibit leanness and weight loss due to hypophagia and increased energy expenditure in mice (Shimada et al., 1998; Kokkotou et al., 2005; Alon and Friedman, 2006; Izawa et al., 2022). On the same line, Jeon et al. (2006) confirmed the leaned phenotypes in aged mice lacking the *Mch* gene, but also reported better glucose tolerance and insulin sensitivity in these animals. Moreover, mice lacking MCH receptor 1 (MCH-R1) present normal body weight, and lean phenotype with decreased fat mass because of their hyperactivity. Intriguingly, MCH-R1 deficient mice are hyperphagic when fed on a regular chow diet and substantially resistant to high-fat diet induced obesity (Marsh et al., 2002). Furthermore, the hyperphagic phenotype persisted in *ob*/*ob* mice lacking the *Mch* gene, however, they exhibit a remarkable reduction in body fat due to increased energy expenditure. Regarding glucose homeostasis, the disruption of the *Mch* gene in ob/ob mice has improved glucose tolerance but hyperinsulinemia remained (Segal-Lieberman et al., 2003). It is noteworthy that insulin increases the activity of LH_MCH_ neurons through phosphatidylinositol 3-kinase signaling. Thus, it has been revealed that specific deletion of insulin receptors in LH_MCH_ neurons does not affect energy balance and glucose homeostasis in mice fed on a regular chow diet whereas it improved peripheral glucose metabolism by enhancing hepatic insulin sensitivity and suppressing the production of hepatic glucose in mice exposed to high-fat diet (Hausen et al., 2016). In aggregate, the abovementioned data (summarized in Table 1) indicate that LH_MCH_ neurons are fundamental in regulating energy expenditure and glucose homeostasis, therefore they might be an attractive target for innovative and efficient treatment for obesity and its comorbidities. The mechanistic signaling of LH_MCH_ neurons implicated in regulating energy expenditure and glucose metabolism is not clearly understood yet. Recently, Izawa et al. (2022) suggested that LH_MCH_ neurons might regulate brown adipose tissue (BAT) activity and energy expenditure by sending projections to the medullary raphe nucleus to inhibit sympathetic inputs in BAT. Besides the traditional synaptic transmission, LH_MCH_ neurons convey its orexigenic effects through a complementary pathway involving the cerebral spinal fluid (CSF). It has been shown that LH_MCH_ neurons modulate CSF flow by regulating the frequency of ciliated ependymal cells in the third ventricle (Conductier et al., 2013). Additionally, chemogenetic activation of LH_MCH_ neurons triggers the release of MCH peptide into the CSF which in turn promotes an increment in food intake whereas the limitation of the bioavailability of MCH present in the CSF significantly reduced feeding (Noble et al., 2018). Importantly, recent outcomes unveiled that LH_MCH_ neurons expressing the vascular endothelial growth factor A (VEGFA) regulate the permeability of the median eminence (ME) microvascular plexus and, thus, modulate leptin action in the arcuate nucleus (ARC) to control food intake through VEGFA-dependent mechanism (Jiang et al., 2020). Together, these data propose the functional interaction between LH_MCH_ neurons and ME barrier components in sensing and processing circulating metabolic signals fundamental to regulating energy homeostasis and metabolism.

**TABLE 1:** Summary of studies that investigated the role of MCH system in food intake and metabolism.

Experiment	Species	Food intake	Plasma glucose level	Plasma insulin level	Plasma leptin level	References
Acute ICV infusion of MCH	Rats (long-Evans)	Increased (regular diet)	–	–	–	Qu et al., 1996 Sahu, 1998 Della-Zuana et al., 2002
	Rats (Sprague-Dawley)	Increased (regular diet)	–	–	–	
	Rats (Wistar; Sprague-Dawley)	Increased (regular diet)	–	–	–	
Chronic ICV infusion of MCH	Rats (Wistar; Sprague-Dawley)	Increased (regular diet)	–	–	–	Della-Zuana et al., 2002 Gomori et al., 2003; Glick et al., 2009 Gomori et al., 2003; Ito et al., 2003
	Mice (C57BL/6J)	Slight increase (regular diet)	No significant changes (regular diet)	No significant changes (regular diet)	Increased (regular diet)	
	Mice (C57BL/6J)	Increased (moderate high fat diet)	Increased (moderate high fat diet)	Increased (moderate high fat diet)	Increased (moderate high fat diet)	
Genetic overexpression of MCH	Transgenic mice (FVB MCH-OE)	Increased (high fat diet)	Increased (High fat diet)	Increased (high fat diet)	Increased (high fat diet)	Ludwig et al., 2001
*Mch* gene knockout	Transgenic mice (*Mch*^–/–^)	Decreased (regular diet)	No significant changes (regular diet)	No significant changes (regular diet)	Decreased (regular diet)	Shimada et al., 1998 Segal-Lieberman et al., 2003 Kokkotou et al., 2005 Jeon et al., 2006
	Transgenic mice (*Mch*^–/–^/*ob/ob*)	Hyperphagic (regular diet)	Decreased (regular diet)	Increased (regular diet)	–	
	Transgenic mice (*Mch*^–/–^/C57BL/6J)	No significant changes (regular diet and high fat diet)	No significant changes (regular diet) Decreased (high fat diet)	No significant changes (regular diet) Decreased (high fat diet)	No significant changes (regular diet) Decreased (high fat diet)	
	Transgenic mice (*Mch*^–/–^/129)	Increased (regular diet and high fat diet)	No significant changes (regular diet) Decreased (high fat diet)	No significant changes (regular diet and high fat diet)	No significant changes (regular diet) Decreased (high fat diet)	
	Transgenic mice (Aged *Mch*^–/–^)	No significant changes (regular diet)	Decreased (regular diet)	Decreased (regular diet)	–	
MCH neurons ablation	Transgenic mice (MCH/ataxin-3)	Decreased (regular diet)	Decreased (regular diet)	Decreased (regular diet)	Decreased (regular diet)	Alon and Friedman, 2006 Izawa et al., 2022
	Transgenic mice (MCH/ataxin-3/*ob/ob*)	–	Decreased (regular diet)	No significant changes (regular diet)	No significant changes (regular diet)	
	Transgenic mice (*MCH-tTA; TetO-DTA*)	No significant changes (regular diet)	–	–	–	
MCH1-receptors deletion	Transgenic mice (*Mch1r*^–/^*^–^*)	Increased (regular diet) Decreased (high fat diet)	No significant changes (regular diet)	No significant changes (regular diet)	Decreased (regular diet)	Marsh et al., 2002
Insulin receptors inactivation on MCH neurons	Transgenic mice IR^Δ^ ^MCH^ mice	No significant changes (regular diet and high fat diet)	No significant changes (regular diet) Improved pyruvate tolerance (high fat diet)	No significant changes (regular diet) Improved insulin sensitivity (high fat diet)	No significant changes (regular diet and high fat diet)	Hausen et al., 2016
Chemoactivation of MCH neurons	Rats (Sprague-Dawley)	Increased (regular diet)	–	–	–	Noble et al., 2018

## LH_MCH_ neurons and sleep-wake cycle

There is numerous evidence that establishes the role of LH_MCH_ neurons in sleep-wake regulation. Based upon the earlier neuroanatomical experiments where c-Fos was used as a marker of neuronal activity, Verret et al. (2003) noticed that a large majority of LH_MCH_ neurons were active during rapid eye-movement (REM) sleep rebound that followed 72 h of selective REM sleep deprivation. In addition, they found that ICV infusions of MCH peptide significantly increased the number of REM bouts (up to 200%) without affecting their duration and provoked a modest prolongation in the time spent in non-rapid eye-movement (NREM) sleep (up to 70%) in a dose-dependent manner (Verret et al., 2003). Subsequent histological studies supported the previous findings and reinforced the hypothesis implying LH_MCH_ neurons in promoting sleep (Modirrousta et al., 2005; Hanriot et al., 2007; Kitka et al., 2011). Similarly, other studies explore the effects of microinjections of MCH into different brain areas involved in sleep-wake regulation. Of note, targeted injections of MCH into wake-promoting nuclei including the dorsal raphe nucleus (DRN) in the rat and cat (Lagos et al., 2009; Devera et al., 2015), median raphe nucleus (MnR) (Pascovich et al., 2020, 2021), LC (Monti et al., 2015), BF (Lagos et al., 2012), and nucleus pontis oralis (NPO) of the cat (Torterolo et al., 2009) produced a dose-dependent increase in REM sleep. In contrast, the local infusions of MCH into the sublaterodorsal tegmental nucleus (SLD), recognized as the key structure involved in REM sleep generation, significantly impeded REM sleep in rats because of decreasing the time spent in REM sleep and the number of REM bouts (Monti et al., 2016). Moreover, microinjections of MCH peptide directly into the VLPO, one of the major NREM-promoting regions, increased the time spent in NREM sleep without affecting REM sleep (Benedetto et al., 2013). Curiously, subcutaneous administration of MCH-R1 antagonists decreased the time spent in sleep stages and prolonged the onset latency of both NREM and REM sleep (Ahnaou et al., 2008), however, the oral supply of MCH-R1 antagonist has no effects on sleep-wake pattern (Able et al., 2009). In agreement with the pharmacological studies, MCH-R1 knockout mice exhibit a significant decrease in NREM sleep through the light-dark cycle, along with this an enhancement in wakefulness and reduction of REM sleep was detected in these transgenic mice when they were exposed to a restraint stress procedure, followed by a homeostatic rebound sleep (Ahnaou et al., 2011). Moreover, targeted deletion of the *Mch* gene in mice increased wakefulness and reduced time spent in NREM and REM sleep compared to wild-type animals. Under fasting conditions, these transgenic mice displayed a massive reduction in REM sleep and remarkable hyperactivity correlated with their lean phenotype (Willie et al., 2008). These behavioral responses in mice lacking the *Mch* gene drew the attention of researchers to investigate MCH-dependent mechanisms underlying sleep-wake regulation in response to changes in energy homeostasis (Willie et al., 2001; Arrigoni et al., 2019). Inconsistent with the previous reports which depicted the integral role of the MCH system in REM sleep regulation, Adamantidis et al. (2008) showed that MCH-R1 knockout mice present an unexpected increase in the REM sleep during the natural sleep-wake cycle and after total sleep deprivation. This differing outcome might be related to compensatory mechanisms established to counterbalance the MCH-R1 disruption caused by the targeted gene deletion approach. Consistent with these crucial findings, *in vivo* electrophysiology recordings combined with juxtacellular labeling of neurons in head-fixed rats revealed that LH_MCH_ neurons were quiet during wakefulness, occasionally firing during NREM sleep and discharging at their maximum rate during REM sleep (Hassani et al., 2009). Subsequently, both deep-brain calcium imaging and fiber photometry studies performed in freely behaving mice have also confirmed that LH_MCH_ neurons displayed a robust activity during REM sleep as well as during the transition from NREM to REM sleep, whereas there were less active in wakefulness and during the transition from REM sleep to wakefulness (Blanco-Centurion et al., 2019; Izawa et al., 2019). Despite this outstanding experimental evidence, no consensus has been reached yet to decipher the specific role of LH_MCH_ neurons in the regulation of REM and NREM sleep. To clarify this point, a collection of optogenetic or chemogenetic experiments were deployed to scrutinize the defined role of LH_MCH_ neurons in the neurobiological mechanisms of sleep-wake behavior. For instance, acute optogenetic activation of LH_MCH_ neurons at 20 Hz during NREM sleep enhanced transitions from NREM to REM sleep while the duration of REM sleep episodes was significantly extended when the optogenetic stimulation of LH_MCH_ neurons occurred at the onset of REM sleep (Jego et al., 2013). Another group of researchers showed that optogenetic stimulation of LH_MCH_ neurons at 10 Hz for 3 h facilitated the transition from NREM to REM sleep, resulting in a significant increase in the time spent in REM sleep and a concomitant decrease in NREM sleep time (Tsunematsu et al., 2014). Surprisingly, chronic optogenetic activation of MCH neurons (ZI and LH) induced a robust increase in the total time in NREM and REM sleep during the night period and notably increased electroencephalogram (EEG) delta power (0.5–4 Hz), an electrophysiological indicator of sleep intensity. For note, Konadhode et al. (2013) and Blanco-Centurion et al. (2016) found that only REM sleep time extended upon optogenetic stimulation during the daytime in nocturnal rodents. Presumably, these divergent outcomes reported in the abovementioned optogenetic studies might be due to differences in genetic strategies implemented to selectively target MCH neurons as well as to differences in the light pulse stimulation paradigms applied to manipulate the activity of these neurons. In addition, chemogenetic activation of LH_MCH_ neurons increased the number of REM bouts during the light period, which was doubled during the dark period, whereas the duration of bouts did not change (Vetrivelan et al., 2016). In this study, the authors demonstrated that LH_MCH_ neurons play a crucial role in promoting REM sleep and facilitating transitions from NREM to REM sleep. Nevertheless, the role of LH_MCH_ neurons in the spontaneous REM sleep generation is still perplexing since several experiments have yielded inconsistent outcomes. For instance, selective ablation of MCH neurons using a genetically targeted diphtheria toxin approach significantly increased the number of REM bouts and shortened the mean bout duration of wake during the light period (Vetrivelan et al., 2016). In another study, Tet-Off system mice were used to specifically disrupt MCH neurons in a reversible and controlled manner. Paradoxically, the results obtained from this study showed that MCH neurons ablation did not affect the total time in REM sleep and the mean episode duration of REM sleep (Tsunematsu et al., 2014). Moreover, transgenic mice with Ataxin 3-mediated ablation of MCH neurons unexpectedly displayed an increase in the REM sleep amounts during the light period (Varin et al., 2016). Conversely, acute optogenetic silencing of MCH neurons, while mice were in REM sleep, did not change REM sleep episode duration (Jego et al., 2013). In addition, chemogenetic inhibition of MCH neurons increased the NREM sleep amounts and notably extended the mean bout duration of NREM sleep without affecting REM sleep duration, suggesting that active MCH neurons hinder the generation of NREM sleep to facilitate the entry into REM sleep (Varin et al., 2018).

Collectively, evidence from pharmacological, electrophysiology, genetic, chemogenetic, and optogenetic studies (summarized in Table 2) revealed that the MCH system plays a critical role in the regulation of REM sleep, whereas further investigations are required to unravel their role in NREM sleep modulation.

**TABLE 2:** Summary of studies that investigated the role of MCH system in sleep-wake regulation.

Experiment		Species	Effect on wakefulness	Effect on NREM sleep	Effect on REM sleep	References
Acute ICV infusion of MCH		Rats (Sprague-Dawley)	Decreased in wake amounts	Increased in NREM amounts	Increased in REM amounts Increased in the number of REM bouts No change in the duration of REM bouts	Verret et al., 2003
Microinjection of MCH	DRN	Rats (Wistar)	Decreased in wake amounts	Moderate increase in the NREM amounts	Increased in REM amounts Increased in the number of REM bouts No change in the duration of REM bouts	Lagos et al., 2009
	MnR	Rats (Wistar)	Decreased in wake amounts	No significant change in NREM amounts	Increased in REM amounts Increased in the number of REM bouts No change in the duration of REM bouts	Pascovich et al., 2021
	LC	Rats (Wistar)	No significant change in wake amounts	No significant change in NREM amounts	Increased in REM amounts Increased in the number of REM bouts No change in the duration of REM bouts	Monti et al., 2015
	BF	Rats (Wistar)	Decreased in wake amounts during the first 2-h post-injection	No significant change in NREM amounts	Increased in REM amounts during the first 2-h post-injection Increased in the number of REM bouts No change in the duration of REM bouts	Lagos et al., 2012
	NPO	Cats	Decreased in wake amounts during the first hour post-injection	No significant change in NREM amounts	Increased in REM amounts during the first hour post-injection No change in the number of REM bouts No change in the duration of REM bouts Decreased in the latency to REM	Torterolo et al., 2009
	SLD	Rats (Wistar)	No significant changes in wake amounts	No significant change in NREM amounts	Decreased in REM amounts during the first and the second 2-h post-injection Decreased in the number of REM bouts No change in the duration of REM bouts Increased in the latency to REM	Monti et al., 2016
	VLPO	Rats (Wistar)	Decreased in wake amounts during 4–5 h block post-injection Decreased in the duration of Wake bouts	Increased in NREM amounts	No change in REM amounts	Benedetto et al., 2013
Pharmacological blockade of MCH-R1	Subcutaneous administration	Rats (Sprague Dawley)	Increased in wake amounts Increased in the number of wake bouts Moderate increase in the duration of Wake bouts at the higher dose	Decreased in NREM amounts Decreased in the duration of NREM bouts	Decreased in REM amounts Decreased in the number of REM bouts Decreased in the duration of REM bouts at the higher dose	Ahnaou et al., 2008
	Oral administration		No change in wake parameters	No change in NREM parameters	No change in REM parameters	Able et al., 2009
MCH1-receptors deletion		Transgenic mice (*Mch1r*^–/^*^–^*)	No change in wake parameters	No change in NREM parameters	Increased in REM amounts during light period Increased in the number of REM bouts during the light period	Adamantidis et al., 2008
			Increased in wake amounts Increased in the duration of wake bouts No change in the number of wake bouts Increased in wake amounts under restraint stress condition	Decreased in NREM amounts Decreased in the duration of NREM bouts No change in the number of NREM bouts Decreased in NREM amounts under restraint stress condition	No change in REM parameters Decreased in REM amounts under restraint stress condition	Ahnaou et al., 2011
*Mch* gene knockout		Transgenic mice (*Mch*^–/–^)	Increased in wake amounts Increased in the duration of wake bouts Increased in wake amounts under fasting condition during both light and dark phase Increased in the duration of wake bouts during both light and dark phase	Decreased in NREM amounts Decreased in NREM amounts under fasting condition during both light and dark phase	Decreased in REM amounts Massive decrease in REM amounts under fasting condition during both light and dark phase Decreased in the duration of REM bouts under fasting condition during both light and dark phase	Willie et al., 2008
*In vivo* electrophysiology (Unit recordings of MCHergic neurons)		Rats (long-Evans)	MCHergic neurons not firing	MCHergic neurons fired occasionally	MCHergic neurons fired maximally	Hassani et al., 2009
Optogenetic manipulation of MCH neurons	Acute activation of MCH neurons at the onset of NREM	Transgenic mice (*Pmch*-*Cre*)	–	No change in the duration of NREM bouts Increased in the transition from NREM-to-REM	–	Jego et al., 2013
	Acute activation of MCH neurons at the onset of REM			–	Increased in the duration of REM bouts	
	Inhibition of MCH neurons at the onset of REM			No changes	Decreased in the frequency and amplitude of REM theta power	
	Acute activation of MCH neurons	*MCH-tTA; TetO ChR2* bigenic mice	No change in wake amounts Increased in the number of wake bouts	Decreased in NREM amounts Decreased in the duration of NREM bouts Increased in the number of NREM bouts Increased in the transition from NREM-to-REM	Increased in REM amounts Increased in the number of REM bouts	Tsunematsu et al., 2014
	Acute inhibition of MCH neurons	*MCH-tTA; TetO ArchT* bigenic mice	No changes	No changes	No changes	
	Chronic activation of MCH neurons	C57BL/6J mice	Decreased in wake amounts during dark phase Decreased in the duration of wake bouts	Increased in NREM amounts during dark phase No change in the duration of NREM bouts Increased in NREM delta power	Increased in REM amounts during dark phase No change in the duration of REM bouts No change in REM theta power	Konadhode et al., 2013
		Rats (long-evans)	Deceased in wake amounts during dark phase Deceased in the number of wake long bouts (> 32 min) during both day and night phases	Increased in NREM amounts during dark phase Increased in the number of NREM short bouts during both day and night phases Increased in NREM delta power during day phase	Increased in REM amounts during both dark and day phases Increased in the number of REM short bouts during both day and night phases Increased in REM theta power during both night and day phases	Blanco-Centurion et al., 2016
Pharmacogenetic manipulation of MCH neurons	Chemoactivation (0.3 mg/Kg CNO)	Transgenic mice (MCH-Cre)	No changes	No changes	Increased in REM amounts during both day and night phases Increased in the number of REM bouts during both day and night phases No change in the duration of REM bouts	Vetrivelan et al., 2016
	Chemoactivation (0.5 mg/Kg CNO)	Transgenic mice (*Pmch*-*Cre*)	–	Deceased in NREM amounts Decreased in the duration of NREM bouts	Increased in REM amounts Increased in the duration of REM bouts	Varin et al., 2018
	Chemoinhibition (5 mg/Kg CNO)		–	Increased in NREM amounts Increased in the duration of NREM bouts	Deceased in REM amounts No change in the duration of REM bouts	
MCH neurons ablation		Transgenic mice (MCH-Cre/ + ; iDTR)	Decreased in the duration of wake bouts during the day phase	No changes	Increased in REM amounts Increased in the number of REM bouts during the day phase	Vetrivelan et al., 2016
		*MCH-tTA; TetO DTA* bigenic mice	Increased in wake amounts during both light and dark phases No change in the duration of wake bouts during both light and dark phases	Decreased in NREM amounts during both light and dark phases Decreased in the duration of NREM bouts during the dark phase No change on EEG power during NREM	No change in REM amounts in both light and dark phases No change on EEG power during REM	Tsunematsu et al., 2014
		Transgenic mice (MCH/ataxin-3)	No change in wake amounts	No change in NREM amounts	Increase in REM amounts only during the light phase	Varin et al., 2016
Deep brain imaging		Transgenic mice (MCH-Cre)	–	The activity of MCH neurons began to increase during the transition from NREM to REM	Dynamic activation of MCH neurons during REM sleep and exploratory behavior	Blanco-Centurion et al., 2019
Fiber photometry		Transgenic mice (MCH-Cre)	Moderate increase in the activity of MCH neurons	The activity of MCH neurons significantly increased during transitions from NREM to REM and from NREM to Wake	Increased in the activity of MCH neurons The activity of MCH neurons deceased during the transition from REM to Wake	Izawa et al., 2019

## LH_*Orexin*_ neurons

Orexin neurons are exclusively localized in LH and the adjacent perifornical area (PFH) and send widespread projections throughout the central nervous system (CNS) (Peyron et al., 1998; Nambu et al., 1999) implicating in the regulation of various behavioral and physiological processes predominantly associated with feeding behavior, energy homeostasis, sleep-wake cycle, and reward system (Willie et al., 2001; Yamanaka et al., 2002, 2003; Harris et al., 2005). Previous studies demonstrated that orexin neurons produce two excitatory neuropeptides orexin-A and orexin-B (also known as hypocretin 1 and hypocretin 2) (de Lecea et al., 1998; Sakurai et al., 1998) and also co-release glutamate (Torrealba et al., 2003; Henny et al., 2010) as well as the inhibitory neuropeptide dynorphin (Chou et al., 2001) and the inhibitory neurotransmitter GABA (Harthoorn et al., 2005). Additionally, orexin-A and orexin-B depolarize the post-synaptic target membrane resulting in increased neuronal excitability by acting selectively on two G protein-coupled receptors (GPCR) named orexin receptor type 1 (OX1R) and orexin receptor type 2 (OX2R). Interestingly, orexin-A binds to OX1R and OX2R, however, orexin-B binds specifically to OX2R (Sakurai et al., 1998; Ammoun et al., 2003; Scammell and Winrow, 2011). Pieces of evidence from subsequent experiments revealed that OX1R couples exclusively to the G_*q/11*_ subclass of GPCR, whereas OX2R couples to G_*i/o*_ and G_*q*_ subclass of GPCR (Sakurai et al., 1998; van den Pol et al., 1998), mediating orexinergic signaling through the activation of Na^+^/Ca^2+^ exchanger (Eriksson et al., 2001; Yang and Ferguson, 2002; Burdakov et al., 2003), or through the decrease of potassium conductance (Ivanov and Aston-Jones, 2000; Bisetti et al., 2006). Additionally, orexin signaling pathways involved other intracellular mechanisms including the activation of phospholipase D/phosphatidic acid (Johansson et al., 2008), phospholipase A/arachidonic acid (Turunen et al., 2012), and mitogen-activated protein kinase cascade (Ramanjaneya et al., 2009; Wen et al., 2015). It is noteworthy to highlight that the activation of orexin neurons triggers excitatory post-synaptic responses whereas the stimulation of MCH neurons engenders inhibitory post-synaptic effects (Sakurai, 2007; Adamantidis and de Lecea, 2008a).

## LH_*Orexin*_ neurons and energy metabolism

A myriad of investigations in the field of molecular, cellular, and behavioral neuroscience provide evidence suggesting the implication of orexin neurons in the regulation of feeding behavior and energy homeostasis. Several studies established that orexin system dysfunction has been implicated in serious neurological disorders including narcolepsy (Lin et al., 1999; Thannickal et al., 2000), addiction (Georgescu et al., 2003; Boutrel et al., 2005; Espana et al., 2011), depression (Taheri et al., 2001; Allard et al., 2004; Brundin et al., 2007; Rotter et al., 2011), anxiety (Suzuki et al., 2005; Li et al., 2010; Avolio et al., 2011; Lungwitz et al., 2012), post-traumatic disorder (Strawn et al., 2010), schizophrenia (Nishino et al., 2002; Dalal et al., 2003; Meerabux et al., 2005; Fukunaka et al., 2007), and severe eating behaviors and metabolic impairments such as anorexia nervosa (Bronsky et al., 2011; Janas-Kozik et al., 2011), hyperphagia and eventually obesity in Prader-Willi syndrome (Nevsimalova et al., 2005; Fronczek et al., 2009).

Several studies have reported that prepro-orexin mRNA level and also the activity of orexin neurons was significantly increased during fasting (Sakurai et al., 1998; Lopez et al., 2000; Diano et al., 2003; Horvath and Gao, 2005). Further, ICV microinjection experiments of orexins have confirmed the potential role of orexins in feeding behavior and energy homeostasis. In fact, acute ICV administration of orexin-A in freely fed rats enhanced food consumption in a dose-dependent manner during the light phase (Sakurai et al., 1998; Edwards et al., 1999; Haynes et al., 1999; Dube et al., 2000; Jain et al., 2000; Yamanaka et al., 2000; Lopez et al., 2002). In the same line with these previous outcomes, microinjections of orexin-A directly into different hypothalamic nuclei such as paraventricular nucleus (PVN), dorsomedial nucleus (DMN), LH, and PFH significantly increased food intake, however, no effect was detected after performing microinjections into ARC, ventromedial nucleus (VMN), preoptic area (POA), central nucleus of the amygdala (CeA) and nucleus of the tractus solitaries (NTS). Similar experiments showed that orexin-B failed to stimulate feeding behavior after infusing it into the aforementioned brain areas (Dube et al., 1999; Thorpe et al., 2003). However, Sweet et al. (1999) reported that orexin-B stimulated feeding only after ICV administration. Subsequent pharmacological studies established that the blockade of OX1R by a selective antagonist (SB-334867-A) provokes a robust reduction in food intake in both fed and fasted rats (Haynes et al., 2000; Rodgers et al., 2001; White et al., 2005). Recently, Jin et al. (2020) reported that infusion of orexin-A into the CeA robustly enhanced palatable high-fat diet consumption suggesting a possible role of the orexin system in the regulation of hedonic feeding. Surprisingly, microinjection of orexin-A into the VLPO significantly increased spontaneous physical activity and non-exercise thermogenesis without affecting food consumption resulting in body weight loss, while blockade of OX1R and OX2R abolished the aforesaid effects of orexin-A and presumably lead to body weight gain (Mavanji et al., 2015; Coborn et al., 2017). These pharmaceutical and behavioral studies endeavoring to elucidate the role of the orexin system in feeding behavior and energy homeostasis are also supported by implementing genetic approaches. Mice lacking orexin neurons (orexin/ataxin-3 transgenic mice) or orexin gene (prepro-orexin knockout mice) exhibit a significant reduction in food intake, water intake, locomotor activity, energy expenditure, and unexpectedly late-onset obesity despite their hypophagic phenotype (Hara et al., 2001, 2005; Fujiki et al., 2006; Zhang et al., 2007). Likewise, selective ablation of orexin neurons using diphtheria toxin fragment A reduced food intake and water intake in orexin-Cre mice even though their body weight was significantly higher compared with control mice (Inutsuka et al., 2014). In agreement with these findings, chemogenetic activation of orexin neurons leads to a robust increase in food intake, water intake, spontaneous physical activity, and the respiratory exchange ratio (Inutsuka et al., 2014). Conversely, another study showed that pharmacogenetic activation of orexin neurons produced a strong enhancement in spontaneous physical activity concomitant with an increase in energy expenditure and unpredictably without inducing any changes in food intake and water intake (Zink et al., 2018). Collectively, these findings (summarized in Table 3) suggest that orexins and their receptors might be considered a promising therapeutic target for the treatment of eating disorders and energy metabolism disturbances including obesity, diabetes, and cardiovascular diseases.

**TABLE 3:** Summary of studies that investigated the role of orexin system in food intake and metabolism.

Experiment			Species	Effect on food intake	References
Acute ICV infusion of orexins		Orexin-A	Freely fed rats (Wistar and Sprague-Dawley)	Increased food intake during the light phase	Sakurai et al., 1998; Edwards et al., 1999; Sweet et al., 1999; Dube et al., 2000; Jain et al., 2000; Yamanaka et al., 2000; Lopez et al., 2002
			Freely fed rats (Wistar and Sprague-Dawley)	Increased food intake during the early light phase (first 4-h post-infusion) Failed to stimulate feeding when given prior the onset of darkness Increased feeding when given 6h into the dark phase	Haynes et al., 1999
			Freely fasted (18 h) rats (Wistar and Sprague-Dawley)	Increased food intake during the first 4-h post-infusion and reduced during the next 20-h	
		Orexin-B	Freely fed rats (Wistar and Sprague-Dawley)	Increased food intake during the light phase	Sakurai et al., 1998; Edwards et al., 1999; Jain et al., 2000
				Failed to stimulate feeding during the early light phase	Haynes et al., 1999
Chronic ICV infusion of orexins		Orexin-A	Freely fed rats (Wistar and Sprague-Dawley)	Increased food intake during the light phase Decreased food intake during the dark phase	Haynes et al., 1999
		orexin-B		Failed to stimulate feeding during the early light phase	
Microinjection of orexins	PVN	Orexin-A	Rats (Sprague-Dawley)	Increased food intake	Dube et al., 1999
	DMN				
	LH	Orexin-A	Rats (Sprague-Dawley)	Increased food intake	Dube et al., 1999; Sweet et al., 1999; Thorpe et al., 2003
	PFH	Orexin-A	Rats (Sprague-Dawley)	Increased food intake	Dube et al., 1999; Sweet et al., 1999
	ARC	Orexin-A	Rats (Sprague-Dawley)	Failed to stimulate feeding	Dube et al., 1999
	VMN				
	POA				
	CeA				
	NTS				
	VLPO	Orexin-A	Rats (Sprague-Dawley)	Failed to stimulate feeding Increased spontaneous physical activity Increased non-exercise activity thermogenesis Stimulate body weight loss	Mavanji et al., 2015; Coborn et al., 2017
	PVN	Orexin-B	Rats (Sprague-Dawley)	Failed to stimulate feeding	Dube et al., 1999; Sweet et al., 1999
	DMN				
	LH				
	PFH				
	ARC				
	VMN				
	POA				
	CeA				
Pharmacological blockade of OX-Rs		OX1R antagonist (intraperitoneal administration)	Freely fed rats (Sprague-Dawley and Lister hooded)	SB-334867-A given during the light phase decreased orexin-A-induced feeding	Haynes et al., 2000; Rodgers et al., 2001
			Freely fasted (18 h) rats (Sprague-Dawley)	SB-334867-A given during the light phase reduced food intake during the first 4-h after overnight fasting	
			Freely fed rats (Sprague-Dawley)	SB-334867-A given during early dark phase reduced food intake during the next 24-h post-injection SB-334867-A given for 3 days during early dark phase reduced food intake over 24-h on days one and three	
			Freely fed rats (Osborne-Mendel and S5B/PI)	SB-334867-A given during early dark phase reduced food intake in both strain fed at high-fat diet but only in Osborne-Mendel fed at low-fat diet SB-334867-A given early dark phase decreased body weight only in the Osborne-Mendel but not in the S5B/PI	White et al., 2005
		OX2R antagonist (in VLPO)	Freely fed rats (Sprague-Dawley)	JNJ-10397049 given during early light phase reduced spontaneous physical activity	Mavanji et al., 2015
		OX1R and OX2R antagonist (in VLPO)	Freely fed rats (Sprague-Dawley)	TCS-1102 (selective dual orexin receptors antagonist) given during early light phase decreased the effect of orexin-A on spontaneous physical activity and energy expenditure	Coborn et al., 2017
Orexin neurons ablation			Transgenic mice (orexin/ataxin-3)	Decreased in food intake Decreased in water intake Decreased in locomotor activity Decreased energy expenditure Mice showed late-onset obesity Increased in the leptin level in females	Hara et al., 2001; Hara et al., 2005; Fujiki et al., 2006; Zhang et al., 2007
			Transgenic mice (orexin-Cre) selective ablation of orexin neurons using diphtheria toxin fragment A	Decreased food intake Decreased water intake Increased body weight Decreased blood glucose level No change in locomotion	Inutsuka et al., 2014
*Orexin* gene knockout			Transgenic mice (prepro-orexin knockout mice with C57/BL6J background)	Male mice showed a mild tendency to late-onset obesity	Hara et al., 2005
			Transgenic mice (prepro-orexin knockout mice with mixed genetic background C57/BL6J and 129SvEv)	Female mice showed more prominent late-onset obesity Increased in the leptin level in females	Fujiki et al., 2006
Chemoactivation of orexin neurons			Transgenic mice (orexin-Cre)	Increased food intake Increased water intake Increased locomotor activity Increased the respiratory exchange ratio Increased blood glucose independently from food intake	Inutsuka et al., 2014
			Transgenic mice (orexin-Cre)	Increased spontaneous physical activity No change in food intake No change in water intake Increased energy expenditure especially in mice fed at high-fat diet	Zink et al., 2018

## LH_*Orexin*_ neurons and sleep-wake cycle

The orexin neurons are wake-active neurons that fire during the wake period and the extracellular level of orexin peak during wakefulness (Kiyashchenko et al., 2002; Lee et al., 2005) and remain silent during NREM and REM sleep with the exception of burst discharge in phasic REM (Mileykovskiy et al., 2005). It has been shown that ICV injection of orexin induces long periods of wakefulness and suppresses the NREM period (Mieda et al., 2011). Both chemogenetic and optogenetic stimulation of orexin neurons produce wakefulness and strongly suppress REM sleep (Adamantidis et al., 2007; Sasaki et al., 2011). It is argued that the most essential role of orexin is to maintain wakefulness (summarized in Table 4). For example, selective loss of orexin neurons in humans causes narcolepsy (Blouin et al., 2005). The deletion of OX2R produces a phenotype like narcolepsy and restoration of OX2R in double knock-out mice rescues normal sleep-wake phenotype in the mice (Willie et al., 2003; Mochizuki et al., 2011). These experiments suggest that OX2R signaling is crucial for controlling sleep-wake.

**TABLE 4:** Summary of studies that investigated the role of orexin system in sleep-wake regulation.

Experiment		Species	Effect on arousal	References
Acute ICV infusion of orexins	Orexin-A	Wild type mice (C57BL/6J), Rats (Sprague-Dawley and hooded lister)	Increased in wake amounts Decreased in NREM and REM amounts cycle Increased in locomotor activity	Hagan et al., 1999; Piper et al., 2000; Espana et al., 2002; Mieda et al., 2011
*Orexin* gene knockout		Transgenic mice (*orexin* -/- with C57BL/6J-129/SvEv mixed background)	Increased in the number of NREM and REM bouts during the dark phase Decreased in the duration of NREM and REM bouts during the dark phase Decreased in REM latency during the dark phase Decreased in the duration of wake bouts during the dark phase Alterations in the circadian frequencies of REM episodes Increased fragmentation of the sleep-wake cycle Hypersomnia	Chemelli et al., 1999; Willie et al., 2003; Mochizuki et al., 2004
Orexin neurons ablation		Transgenic mice (orexin/ataxin-3)	Increased in REM amount during the dark phase Increased in the duration of REM bouts during the dark phase Decreased in the duration of wake bouts during the dark phase Increased fragmentation of the sleep-wake cycle	Hara et al., 2001; Zhang et al., 2007
OX2-receptor deletion		Transgenic mice (*OX2R*^–/–^) OX2R Transcription-Disrupted mice	Increased fragmentation of the sleep-wake cycle Decreased in the duration of wake during the dark phase Decreased in the duration of NREM during the dark phase Decreased in REM latency during the dark phase	Willie et al., 2003; Mochizuki et al., 2011
Optogenetic stimulation of orexin neurons		Transgenic mice (*Hcrt:EGFP*) injected with lentivirus Hcrt:ChR2-mCherry	Increased the transition to wake from NREM or REM 5–30 Hz light pulse trains decreased wake latency Strong reduction of REM duration	Adamantidis et al., 2007
Chemoactivation of orexin neurons		Transgenic mice (orexin-Cre)	Increased in wake amounts during the light phase Decreased in NREM amounts during the light phase Decreased in REM amounts during the light phase Modest increase in wake amounts during the dark phase Increased in the latency from wake to REM during the dark phase	Sasaki et al., 2011

There are conflicting results reported regarding histamine as a signaling element in orexin actions. Carter et al. (2009) have shown that optogenetic stimulation of orexin neurons promotes arousal in mice lacking central histamine. Contrary to this, central administration of orexin induces wakefulness in wild-type animals but not in histamine receptor 1 knock-out mice. Orexin neurons also influence sleep as it reduces the NREM and REM episodes. The central orexin signaling results in reduced REM sleep duration (Williams et al., 2008; Mieda et al., 2011; Sasaki et al., 2011). This effect is possibly mediated by the activation of both OX1R and OX2R (Mieda et al., 2011). Narcoleptic patients show short latency of REM sleep and random nap often include bouts of REM sleep (Dantz et al., 1994; Andlauer et al., 2013). Like narcoleptic individuals, mice lacking orexin signaling are also unable to suppress REM sleep bouts during the active period, indicating the role of orexin signaling during the active period to suppress REM sleep and meet temporal needs (Arrigoni et al., 2019).

## MCH and orexin neuronal circuitries regulating sleep and metabolism

**FIGURE 1:**
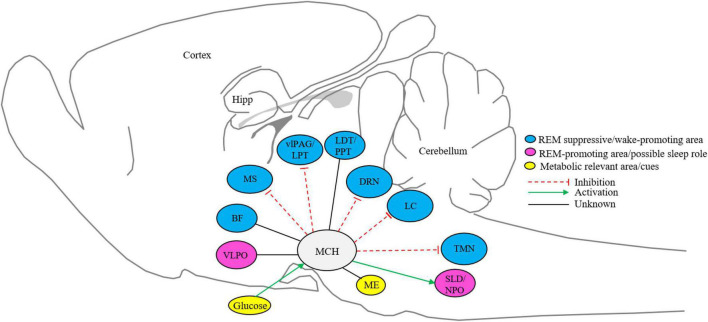
Schematic representation of MCH system. MCH neurons in the lateral hypothalamus and zona incerta project to metabolic relevant and sleep-wake controlling nuclei (Burdakov et al., 2005; Torterolo et al., 2009; Lagos et al., 2012; Benedetto et al., 2013; Jego et al., 2013; Monti et al., 2016; Costa et al., 2018; Kroeger et al., 2019; Jiang et al., 2020). BF, basal Forebrain; DRN, dorsal raphe nucleus; Hipp, hippocampus; ME, median eminence; PPT, pedunculopontine tegmentum; LDT, laterodorsal tegmentum; LC, locus coeruleus; TMN, tuberomammillary nucleus; vLPAG/LPT, ventrolateral periaqueductal gray matter and lateral pontine tegmentum; VLPO, ventrolateral preoptic area; MS, medial septum; SLD/NPO, sublaterodorsal tegmental nucleus and nucleus pontis oralis; MCH, melanin-concentrating hormone neurons. Sleep and metabolic-relevant nuclei are color-coded and excitatory and inhibitory inputs are arrow represented.

The MCH neurons in LH and ZI project to the nuclei that involve in promoting sleep and arousal (Monti et al., 2013). These projections positively modulate sleep, especially REM sleep (Torterolo et al., 2011). The LC, DRN, and regions of the ventrolateral periaqueductal gray matter and lateral pontine tegmentum (vlPAG/LPT) that are implicated in REM sleep regulation receive dense MCH projections (Torterolo et al., 2009; Costa et al., 2018). The activation of the MCH terminal in vlPAG/LPT tends to increase the duration of REM sleep (Kroeger et al., 2019). Overall, MCH neurons promote sleep by inhibiting wake-promoting areas like the medial septum (MS) and TMN (Jego et al., 2013; Figure 1). The SLD within the dorsolateral pons and NPO in the subcoeruleus (anatomical equivalent of the SLD in cats) are characterized as REM promoting area (Torterolo et al., 2009; Luppi et al., 2013). It is conceivable that MCH neurons directly activate SLD or NPO, likely through the release of glutamate (Torterolo et al., 2009, 2013; Monti et al., 2016). Further, it is considered that MCH may interact with REM-promoting cholinergic neurons within LDT and PPT based on the identification of MCH axons in these areas (Costa et al., 2018), however, there are no functional data supporting this circuit (Figure 1).

**FIGURE 2:**
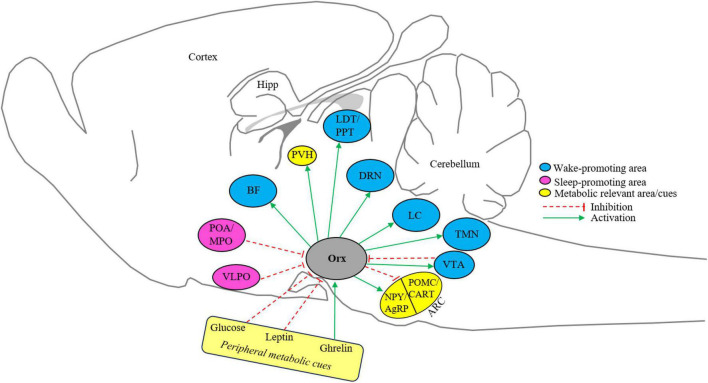
Schematic representation of the orexin system. Orexin neurons in the lateral hypothalamus project and receive projection from metabolic-relevant and sleep-wake-controlling nuclei (de Lecea et al., 1998; Peyron et al., 1998; Date et al., 1999; Horvath et al., 1999b; Brown et al., 2001; Eggermann et al., 2001; Eriksson et al., 2001; Funahashi et al., 2003; Yamanaka et al., 2003; Burdakov et al., 2006; Yoshida et al., 2006; Benedetto et al., 2012; Saito et al., 2013, 2018b; Alam et al., 2014; Baimel et al., 2017; Chung et al., 2017; Chowdhury et al., 2019). BF, basal forebrain; DRN, dorsal raphe nucleus; Hipp, hippocampus; Orx, orexin; POA/MPO, preoptic area/medial preoptic area; LDT, laterodorsal tegmentum; PPT, pedunculopontine tegmentum; PVH, Paraventricular nucleus of the hypothalamus; LC, locus coeruleus; TMN, tuberomammillary nucleus; VLPO, ventrolateral preoptic area; VTA, ventral tegmental area; ARC, arcuate nucleus; NPY, Neuropeptide Y; AgRP, agouti-related protein; POMC, pro-opiomelanocortin; CART, amphetamine-related transcript. Sleep and metabolic-relevant nuclei are color-coded and excitatory and inhibitory inputs are arrow represented.

Orexin neurons are found only in LH and PFH and similarly project in the CNS like MCH neurons, however, having the opposite effect on the modulation of sleep-wake and metabolism (Figure 2). Orexin neurons project to wake-associated neurons in BF, LC, TMN, VTA, and DRN, *in vitro* electrophysiology studies have shown that orexin activates neurons in all these regions (Peyron et al., 1998; Horvath et al., 1999b; Brown et al., 2001; Eggermann et al., 2001; Eriksson et al., 2001; Baimel et al., 2017). So far there is no explicit explanation of the action of orexin neurons on these wake-associated areas, however, a general apprehension is that orexin neurons co-release orexin, dynorphin, and glutamate to likely activate target neurons (Arrigoni et al., 2019). The neurons from sleep-promoting areas like MPO, POA, and VLPO project to orexin neurons in LH, these areas harbor GABAergic neurons that are active during the NREM and/or REM sleep episodes and promote NREM sleep (Yoshida et al., 2006; Benedetto et al., 2012; Saito et al., 2013, 2018a; Alam et al., 2014; Chung et al., 2017). It has been shown that GABA release in LH is higher during the sleep period and blocking GABAergic signaling during the sleeping period activates orexin neurons (Nitz and Siegel, 1996; Alam et al., 2005). Moreover, GABAergic input to LH also reached from VTA as activation of GABAergic neuronal terminals in the LH promoted NREM sleep by inhibiting orexin neurons (Chowdhury et al., 2019). A recent study shows that orexin neurons indirectly target and inhibit sleep-promoting VLPO neurons to promote arousal (De Luca et al., 2022). These findings indicate that sleep-active GABAergic input from preoptic areas inhibits orexin neurons. This GABAergic inhibitions and orexin-mediated activation of wake-associated neurons and inhibition of sleep-associated neurons could be a possible mechanism by which orexin neurons regulate sleep and arousal.

The LH neurons modulate the metabolism by regulating the feeding. The connectivity of LH to ARC may adjust the food intake depending on the energy needs of the animals. The orexin neurons project to ARC that harbor NPY and POMC neurons expressing orexin and leptin receptors (de Lecea et al., 1998; Date et al., 1999; Funahashi et al., 2003). Orexin induces feeding by activating NPY and inhibiting POMC neurons (Dube et al., 2000; Jain et al., 2000; Guan et al., 2001; Ma et al., 2007; Figure 2). The feeding circuity of orexin may extend to PVH as orexin neurons project to PVH and ICV injection of orexin activates ARC (Date et al., 1999; Edwards et al., 1999). However, it is not explicitly known how orexin acts on PVH and whether orexin regulates feeding through PVH. Orexin neurons also act as metabolic sensors as they respond to peripheral metabolic cues. Extracellular glucose inhibits orexin neurons (Burdakov et al., 2006). In addition to that direct sensing of extracellular glucose levels, orexin neurons sense the other peripheral indicators of energy status such as the satiety hormone leptin and hunger hormone ghrelin. Leptin inhibits orexin neurons whereas ghrelin activates the same (Yamanaka et al., 2003). Thus, negative energy balance activates orexin neurons and hence hunger keeps animals awake. Contrary to this, MCH neurons promote positive energy balance. The MCH neurons are activated by high glucose levels and physiological shifts in glucose have the opposite effects on the electrical activity of orexin neurons (Burdakov et al., 2005, 2006). This differential glucose-sensing ability of orexin and MCH neurons suggests that hyperglycemia may reduce feeding by hyperpolarization of excitatory (orexin neurons) and depolarization of inhibitory (MCH neurons) input to ARC neurons. The direct projection of MCH neurons to ARC is not known, however, a recent study suggests that MCH neurons project to the median eminence (ME), and its activation enhances leptin action in the ARC (Jiang et al., 2020).

## Orexin and MCH neurons act as sensors of metabolic changes and arousal

In mammals, maintaining the balance between energy intake and energy expenditure is crucial for survival. However, energy homeostasis imbalance underlies serious metabolic disturbances and diseases such as obesity, diabetes, hyperlipidemia, hypertension, cardiovascular diseases, and cancers (Crowley et al., 2002; Calle et al., 2003; Kim et al., 2016; Tanaka and Itoh, 2019).

**FIGURE 3:**
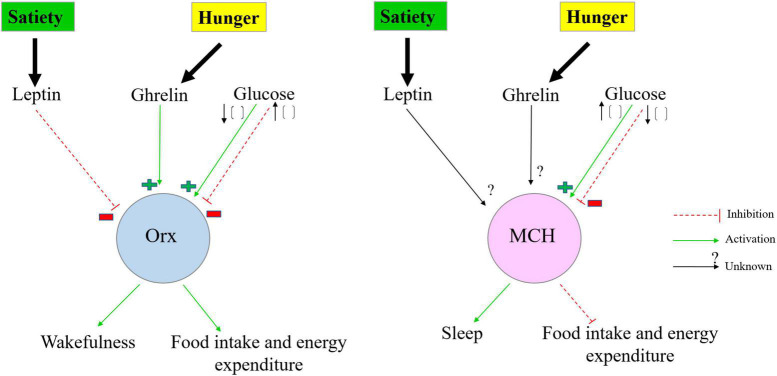
Simplified schematic representation of the orexin and MCH systems as a sensor of metabolic changes and arousal. In negative energy balance, low extracellular glucose concentration and high circulating level of ghrelin activate the orexin system but inhibit MCH neurons leading to an increase in orexin release and a decrease in MCH release to promote wakefulness, activity, foraging, and food intake. By contrast, in positive energy balance, high extracellular glucose concentration activates MCH neurons but suppresses orexin neurons which are also inhibited by the circulating level of leptin promoting sleep and decreasing energy expenditure. Orx, orexin neurons; MCH, melanin-concentrating hormone neurons.

A panoply of experimental evidence revealed that energy homeostasis is regulated via a complex and widespread neuronal circuit located mainly in the brainstem and hypothalamus (Schwartz et al., 2000; Myers and Olson, 2012; Morton et al., 2014; Roh et al., 2016). Distinct neuronal populations within particular nuclei of the brainstem and the hypothalamus sense variations in the energy status of the body by integrating and responding to multiple peripheral (glucose, insulin, leptin, ghrelin, glucagon-like peptide 1) and central [GABA, NPY, AgRP, α-melanocyte-stimulating hormone (α-MSH), serotonin] metabolic signals to maintain energy homeostasis by coordinating energy intake with energy expenditure over time (Burdakov et al., 2005; Schwartz and Porte, 2005; Williams et al., 2008; Morton et al., 2014; Gautron et al., 2015; Roh et al., 2016; Timper and Bruning, 2017; Mavanji et al., 2022). In this context, it is of interest to highlight the crucial role of orexin and MCH systems in regulating energy balance in response to fasting. In fact, the activation of orexin neurons promotes food foraging and increases energy expenditure, whereas the activation of MCH neurons enhances food intake and decreases energy expenditure leading to an increase in energy storage (Figure 3). Both orexin and MCH neurons are activated by fasting (Qu et al., 1996; Sakurai et al., 1998; Lopez et al., 2000; Yamamoto et al., 2000; Diano et al., 2003; Horvath and Gao, 2005; Mogi et al., 2005; Buczek et al., 2020). Interestingly, it was recently revealed that MCH neurons are activated during the early phase of fasting (12 h of fasting), however, orexin neurons exhibit a delayed activation during food deprivation (24 h of fasting). This alternate activation of MCH and orexin neurons play a potential role in coordinating foraging behaviors and energy storage to adjust energy homeostasis during prolonged fasting (Linehan and Hirasawa, 2022). Taken together, these findings insinuate that orexin and MCH neurons are capable of sensing and integrating circulating metabolic signals that convey precise information regarding the status of energy stores, leading to dynamic coordination between energy intake and energy expenditure to restore energy balance.

Pioneering studies reported that orexin-producing neurons are involved in sensing glucose, ghrelin, and leptin levels and eventually promoting arousal (Figure 2). Indeed, electrophysiological evidence revealed that increasing glucose levels induced a striking hyperpolarization and cessation of both spontaneous and evoked action potentials in isolated orexin neurons (Yamanaka et al., 2003; Burdakov et al., 2005; Sheng et al., 2014). Furthermore, the blockade of glycolytic metabolism of glucose by selective inhibitors of glucokinase failed to change the effects of glucose on the action potentials of orexin neurons. These results indicate that orexin neurons are capable to sense trends in glucose levels independently of glucose metabolism (Gonzalez et al., 2008, 2009). Actually, glucose inhibits orexin neurons by acting at the extracellular tandem-pore K + (K_2P_) channels to induce membrane hyperpolarization and decrease the firing rate of orexin neurons (Burdakov et al., 2006). Here, it is worthwhile to highlight that glucose inhibited orexin neurons only when their intracellular energy levels are low, but paradoxically glucose failed to block orexin neurons when the intracellular levels of lactate, pyruvate, and ATP are high. These results reveal an unexpected glucose-sensing mechanism in orexin neurons that is tightly modulated by the cellular energy status (Venner et al., 2011). Strikingly, recent experimental findings showed for the first time an unexpected complex relationship between orexin neuron activity and blood glucose changes in living organisms. In fact, orexin neurons activity vs. blood glucose variability exhibited a non-canonical temporal profile instead of the expected linear pattern. Basically, orexin neurons track blood glucose concentration at the temporal resolution of minutes and promptly convey its changes into targeted brain regions to trigger adaptive behavior strategies in order to optimize energy balance (Viskaitis et al., 2022).

In addition to sensing peripheral glucose changes, orexin neurons are also involved in detecting and processing signals from other circulating factors such as leptin. Leptin, a product of *ob* gene, is an anorexigenic hormone predominantly released by adipose tissues (Zhang et al., 1994) and plays a critical role in regulating satiety, blood glucose levels, and energy homeostasis by acting on defined target neurons of the CNS (Schwartz et al., 1996). Previous findings demonstrated that ICV administration of leptin prevents an increase of prepro-orexin mRNA and orexin receptor 1 mRNA in fasted rats, suggesting that leptin has inhibitory feedback on the regulation of orexin gene expression (Lopez et al., 2000). Moreover, Zhu et al. (2002) confirmed orexin neurons induce feeding behavior through both leptin-sensitive and leptin-insensitive pathways. In this sense, we can speculate that leptin might regulate the activity of orexin neurons via complex circuit mechanisms. Indeed, earlier reports yielded conflicting results concerning the expression of leptin receptors (LepRb) on orexin neurons. Findings from immunohistochemistry studies performed in rodent and monkey brains demonstrated that orexin neurons in the LH possess LepRb and thus supporting the hypothesis that leptin might act directly upon these neurons to reduce food seeking and regulate energy balance (Hakansson et al., 1999; Horvath et al., 1999a; Iqbal et al., 2001). Subsequent investigation revealed that bath application of leptin onto isolated orexin neurons provoked hyperpolarization of the membrane potential and suppressed the action potential firing in these cells, resulting in inhibition of orexin neurons (Yamanaka et al., 2003). However, using transgenic LepRb*^EGFP^* mice where enhanced green fluorescence protein (EGFP) expression is under the control of the LepRb promotor to scrutinize the possible colocalization of EGFP with orexin neurons, displayed that LepRb-expressing neurons represent a distinct population from orexin neurons in the LH (Leinninger et al., 2009; Louis et al., 2010; Laque et al., 2013). In general support of these results, further experimental works were performed using electrophysiology recordings in brain slices, knock-in mice lines and single-cell expression profiling approaches to elucidate that orexin neurons do not express LepRb and are only indirectly regulated by leptin (Leinninger et al., 2011; Goforth et al., 2014; Sheng et al., 2014; Mickelsen et al., 2017). Several studies have shown that LepRb-expressing neurons lie in synaptic contact with orexin neurons within the LH and the majority of these LepRb neurons contain neurotensin (LepRb^Nts^) (Louis et al., 2010; Leinninger et al., 2011). In addition, pharmacogenetic activation of LepRb^Nts^ in hypothalamic slices hyperpolarized membrane potential and reduced action potential firing in orexin neurons. Likewise, the selective genetic deletion of LepRb from LH LepRb^Nts^ neurons abolishes leptin-induced inhibition of orexin neurons (Leinninger et al., 2011; Goforth et al., 2014). Together these data suggest that leptin inhibits indirectly the activity of orexin neurons by acting on LepRb^Nts^ cells within the LH. Here it is worthwhile to emphasize that LepRb^Nts^ also co-release the inhibitory neuropeptide galanin (Laque et al., 2013) which plays an important role in the regulation of orexin neurons by leptin whereas Nts has a tendency to stimulate these cells indicating that this peptide is not implicated in leptin-induced inhibition of orexin neurons (Goforth et al., 2014). It was also reported that leptin failed to significantly enhance GABA_*A*_-mediated inhibitory synaptic transmission in orexin neurons and the blockade of GABA receptors could not prevent leptin inhibition of orexin neurons (Goforth et al., 2014). In aggregate, leptin indirectly inhibits orexin neurons by activating LepRb^Nts^ neurons through the release of galanin and via GABA-independent mechanisms including the presynaptic inhibition of glutamate inputs onto orexin neurons and the post-synaptic opening of ATP-sensitive potassium K_ATP_ channels.

In addition to glucose and leptin, the orexin system is also involved in sensing other circulating factors and hormones such as ghrelin to coordinate behaviors with metabolic needs. Ghrelin is a gastrointestinal hormone released predominantly from the stomach during periods of energy deficit to enhance appetite and food intake (Ariyasu et al., 2001; Nakazato et al., 2001; Lawrence et al., 2002; Olszewski et al., 2003). Importantly, ghrelin is also produced in the brain by a distinct hypothalamic neuronal population adjacent to the third ventricle between the DMH, the VMH, and the ARC. These neurons send wide projections into several hypothalamic nuclei including the ARC and LH to synapse, respectively, with NPY and orexin neurons (Cowley et al., 2003; Toshinai et al., 2003). For note, ghrelin mediates its effects by binding to growth hormone secretagogue receptors, a subtype of the GPCR family highly expressed in the brain as well as in peripheral tissues including stomach, intestine, pancreas, liver, heart, and skeletal muscles (Kojima et al., 1999; Papotti et al., 2000; Gnanapavan et al., 2002; Sun et al., 2004; Yin et al., 2014). Hence, ghrelin can participate in regulating multiple biological processes comprising glucose metabolism (Broglio et al., 2001; Saad et al., 2002; Dezaki et al., 2004; Verhulst and Depoortere, 2012), energy homeostasis (Ravussin et al., 2001; Druce et al., 2005; Malik et al., 2008; Lin et al., 2011), cardiovascular functions (Mao et al., 2012, 2013; Cao et al., 2013; Khazaei and Tahergorabi, 2013), reproduction (Comninos et al., 2014), cell proliferation (Costa et al., 2011; Delhanty et al., 2014; Miao et al., 2019), inflammation and immune system (Lee et al., 2010; Baatar et al., 2011; Wei et al., 2015; Azizzadeh et al., 2017; Santos et al., 2017), learning and memory performance (Carlini et al., 2002; Diano et al., 2006; Kanoski et al., 2013), sleep-wake cycle and (Tolle et al., 2002; Weikel et al., 2003; Szentirmai et al., 2007a,b), and other circadian rhythms (Yannielli et al., 2007; Wang et al., 2018; Qian et al., 2019). Here it is worthwhile to report that ghrelin regulates feeding behaviors and energy homeostasis by interacting with distinct neuronal populations within the CNS including orexin neurons (Olszewski et al., 2003; Toshinai et al., 2003, 2006). In contrast to leptin and glucose, it has been reported that ghrelin stimulates orexin neurons (Yamanaka et al., 2003; Goforth et al., 2014; Sheng et al., 2014). Previous studies have shown that peripheral or central administration of ghrelin robustly increased food intake and induced Fos expression in orexin-immunoreactive neurons but not in MCH-containing neurons (Nakazato et al., 2001; Lawrence et al., 2002; Tolle et al., 2002; Toshinai et al., 2003). Moreover, electrophysiological evidence showed that ghrelin directly activates isolated orexin neurons by inducing membrane depolarization and increasing the action potential firing in these cells (Yamanaka et al., 2003; Sheng et al., 2014). During periods of starvation, elevated circulating levels of ghrelin enhanced the sensitivity of orexin neurons to glucose changes, and thus contribute to maintaining energy homeostasis (Sheng et al., 2014).

In contrast to orexin neurons which are inhibited by glucose, MCH neurons are activated by glucose. A rise in the extracellular glucose levels directly enhanced the excitability of MCH neurons by inducing membrane depolarization of MCH neurons accompanied by an increase in its resistance (Burdakov et al., 2005). Subsequent investigations revealed that glucose sensing by MCH neurons implicates K_ATP_ channels and is modulated by a mitochondrial protein UCP2 that decreases ATP production (Krauss et al., 2003; Kong et al., 2010). Additionally, the action of leptin and ghrelin on MCH neurons is yet to be precisely delineated (Figure 3).

## Perspective

Based on the currently available data, it emerges as orexin and MCH system in LH mediating its opposing action on sleep-wake and energy metabolism by utilizing multiple neuronal circuits and peripheral cues. The behavioral strategy required to regulate arousal with respect to hunger and satiety with respect to sleep is under the control of orexin and MCH neurons and their extended circuitries. The MCH neurons promote sleep whereas orexin promotes wakefulness, whereas both these neurons promote feeding by interacting with ARC neurons. However, the feeding preference is different as orexin neurons motivate palatable food consumption, whereas MCH neurons motivate caloric food consumption. Interestingly, hunger and hypoglycemia activate orexin neurons that induce arousal required for foraging and food consumption. On the other hand, MCH neurons sense the rise in glucose levels and promote inactivity and sleep. This indicates interconnectivity of LH to ARC is crucial in maintaining sleep and energy homeostasis and effective to deal with challenges such as starvation and sleep disruption. Sleep disruption influences metabolic processes (Donga et al., 2010; Jha et al., 2016). Sleep deprivation increases ghrelin and decreases leptin levels (Schmid et al., 2008; Mosavat et al., 2021), which activates the orexin system. Thus, these arousing cues promote consummatory behavior, inhibition of sleep, and energy conservation. The inhibition of this signaling in recovery sleep may stabilize it by maintaining the sleep-wake cycle. How the MCH system senses these metabolic cues are not clear yet, however, the MCH system may respond to it by stabilizing sleep and decreasing the energy expenditure by interacting with the orexin neuron activity and brain circuits involved in sleep and metabolism. Moreover, metabolic abnormalities also disrupt sleep (Ogilvie and Patel, 2017). Disruption of the sleep-wake cycle in obesity and other metabolic conditions is not studied at the mechanistic level. There are possibilities that the peripheral metabolic cues may directly or indirectly interact with the orexin/MCH system to alter the sleep phenotype in metabolic disorders. Metabolic disruption may influence LH’s neuronal systems as it has been shown that obesity shifts the activity and transcriptional profile of LHA glutamatergic neurons (Rossi et al., 2019). By knowing these co-localized and interacting neural systems that govern the distinct and interdependent behavioral programs—sleep and feeding, it would be enticing to dissect the neuronal bases of the interaction of both these behaviors.

## Conclusion

Sleep and the metabolic system are bidirectionally linked to maintaining homeostasis in challenging environments. In this review, we summarized how molecular and cellular components of MCH and orexin signaling maintain this bidirectionality by integration of sleep-wake and energy metabolism. Both these classes of neurons sense the metabolic signals and regulate the sleep-wake states. A substantial chunk of work has been done to understand how orexin and MCH neurons in LH coordinate the metabolism and behavioral states. Future works on how sleep and metabolism influence each other, and the mechanistic explanation of their interaction would be helpful to assign the target for therapeutic intervention for metabolic and arousal-related disorders.

## Author contributions

Both authors listed have made a substantial, direct, and intellectual contribution to the work, and approved it for publication.
